# Adapter-Mediated Transduction with Lentiviral Vectors: A Novel Tool for Cell-Type-Specific Gene Transfer

**DOI:** 10.3390/v14102157

**Published:** 2022-09-30

**Authors:** Nicole Cordes, Nora Winter, Carolin Kolbe, Bettina Kotter, Joerg Mittelstaet, Mario Assenmacher, Toni Cathomen, Andrew Kaiser, Thomas Schaser

**Affiliations:** 1 Miltenyi Biotec B.V. & Co. KG, 51429 Bergisch Gladbach, Germany; 2 Institute for Transfusion Medicine and Gene Therapy, Medical Center-University of Freiburg, 79106 Freiburg, Germany; 3 Center for chronic Immunodeficiency, Faculty of Medicine, University of Freiburg, 79106 Freiburg, Germany

**Keywords:** lentiviral vectors, gene therapy, CAR-T cells, immunotherapy

## Abstract

Selective gene delivery to a cell type of interest utilizing targeted lentiviral vectors (LVs) is an efficient and safe strategy for cell and gene therapy applications, including chimeric antigen receptor (CAR)-T cell therapy. LVs pseudotyped with measles virus envelope proteins (MV-LVs) have been retargeted by ablating binding to natural receptors while fusing to a single-chain antibody specific for the antigen of choice. However, the broad application of MV-LVs is hampered by the laborious LV engineering required for every new target. Here, we report the first versatile targeting system for MV-LVs that solely requires mixing with biotinylated adapter molecules to enable selective gene transfer. The analysis of the selectivity in mixed cell populations revealed transduction efficiencies below the detection limit in the absence of an adapter and up to 5000-fold on-to-off-target ratios. Flexibility was confirmed by transducing cell lines and primary cells applying seven different adapter specificities in total. Furthermore, adapter mixtures were applied to generate CAR-T cells with varying CD4/CD8-ratios in a single transduction step. In summary, a selective and flexible targeting system was established that may serve to improve the safety and efficacy of cellular therapies. Compatibility with a wide range of readily available biotinylated molecules provides an ideal technology for a variety of applications.

## 1. Introduction

Lentiviral vectors (LVs) are a versatile tool for the genetic modification of mammalian cells, as they enable the transduction of dividing or non-dividing cells with sustained gene expression [1,2]. These advantages make LVs attractive for generating chimeric antigen receptor (CAR)-T cell therapies. This promising cancer immunotherapy showed exceptional clinical results, which led to the FDA approval of five therapies for the treatment of leukemia, lymphoma, and myeloma to date [3,4]. State-of-the-art LVs are pseudotyped with the glycoprotein G of vesicular stomatitis virus (VSV-G) [2,5,6]. VSV-G pseudotyped LVs require the expression of the low-density lipoprotein receptor (LDLR) for target cell binding and fusion [7]. As LDLR is expressed by many cell types, VSV-G confers broad tropism to LVs, making the isolation of the desired cell type necessary prior to transduction. Importantly, while many cell types readily express LDLR, T cells require activation to induce upregulation for sufficient transduction [8]. Consequently, the generation of CAR-T cells requires sophisticated ex vivo production protocols starting with T-cell isolation, activation, and gene transfer, followed by an expansion phase [9,10]. Improving this production process could result in a CAR-T cell product with enhanced efficacy. For instance, a shorter ex vivo expansion phase and the transduction of naïve T-cell subsets was shown to increase CAR-T cell efficacy [11,12]. Ruella and colleagues showed that off-target transduction represents a major risk factor in CAR-T cell production, as the transduction of a single leukemic B cell led to resistance to CAR-T cells [13]. Therefore, limiting the gene transfer to the target cell population only may improve the safety and efficacy of a CAR-T cell therapy. 

To date, various strategies were developed to restrict gene transfer to a cell type of interest using cell-type-specific LVs. One promising approach is using LVs pseudotyped with paramyxovirus envelope glycoproteins, e.g., Measles morbillivirus (MV), Nipah virus, or Tupaia paramyxovirus [5,14,15,16]. In contrast to VSV-G LVs, the function of attachment and fusion is separated on two glycoproteins, H or G and the F protein, respectively. To confer specific target cell entry, the viral attachment proteins of the MV and Nipah virus are mutated to abolish binding to their natural receptors. Binding of the attachment protein is restored by fusion of a targeting ligand, such as a single-chain variable fragment (scFv) or a designed ankyrin repeat protein (DARPin) to its C-terminus. MV-LVs are interesting candidates for immunotherapeutic applications, as the efficient and selective transduction of B cells and CD4+ or CD8+ T cells was shown in vitro and in vivo [15,17,18,19,20]. Moreover, MV-LVs were shown to transduce resting T and B cells, making an activation step obsolete [21,22]. A major limitation of this technology is the availability of targeting ligands that are efficiently expressed when fused to the H protein. The success rate of target ligand screening is low, with less than 50% of ligands being efficiently expressed [5]. This time-consuming and costly protein engineering represents a roadblock to the widespread applicability of targeted MV-LVs. In addition, complex applications may require simultaneous transduction of multiple cell types, for which mixing of LVs with different specificities would be needed. To overcome these limitations, a technology is needed that provides the required flexibility while retaining the high standard of selectivity. 

Here, we report the first versatile targeting system for MV-LVs, referred to as Adapter-LV (Ad-LV), which solely requires mixing with biotinylated adapter molecules to enable selective gene transfer. For this, an mBio3-18E7-derived scFv was fused to the H protein that binds biotin in the context of a specific linker [23]. LVs are bound to biotinylated adapter molecules which in turn bind to the antigen of choice on the target cell, thereby conferring specificity. The flexibility of the system was confirmed by transducing cell lines and primary human as well as murine lymphocytes, applying adapters of different specificities. The selectivity was analyzed on co-cultured cell lines and primary cells, revealing a up to 5000-fold on-to-off target ratio. Most importantly, transduction was abolished in the absence of an adapter, providing an additional layer of safety. Adapter molecules of different-format IgG monoclonal antibodies (mAB) and fab or f(ab)_2_ fragments could be used, allowing for the manipulation of size, half-life and avidity. Finally, adapters with different specificities were combined to enable the simultaneous delivery of the CD20-CAR transgene to two cell types, i.e., CD4+ and CD8+ T cells. This way, CAR-T cells with varying CD4/CD8 ratios were generated simply by adjusting the adapter concentrations and specificities. These new features make targeted MV-LVs an attractive tool for a wide range of applications. 

## 2. Materials and Methods

### 2.1. Primary Cells and Cell Lines

HEK-293T and HT1080 cells were cultivated in DMEM (Biowest, Nuaille, France) (10% FCS (Biochrom, Berlin, Germany)). Raji and SupT1 cells were cultured in RPMI (Biowest, Nuaille, France) supplemented with 10% FCS and 2 mM L-glutamine (Biowest, Nuaille, France). PBMCs were isolated from buffy coat derived from healthy donors via density centrifugation using Ficoll (Pan Biotech, Aidenbach, Germany) and cultured in TexMACS™ (Miltenyi Biotec, Bergisch Gladbach, Germany) supplemented with 12.5 ng/mL IL-7 and 12.5 ng/mL IL-15 (both Miltenyi Biotec, Bergisch Gladbach, Germany). For the selectivity assay, PBMCs were cultivated in TexMACS™ supplemented with 50 IU/mL IL-4, 12.5 ng/mL IL-7, and 12.5 ng/mL IL-15 (all Miltenyi Biotec, Bergisch Gladbach, Germany) and 1 µg/mL CD40-ligand (RD Systems, Minneapolis, MN, USA). 

### 2.2. Plasmid Constructs

The glycoproteins H and F in the pCG expression plasmid under the control of the cytomegalovirus promoter have been described previously [15]. For generating the plasmid pCG-H-αBiotin, the scFv coding region in pCG-H-αCD20 was replaced after SfiI/NotI digestion. The α-Biotin scFv was derived from a hybridoma cell line (clone: Bio3-18E7). The sequence of the α-Biotin scFv was aligned to the germline sequence for the identification of unusual residues using the online platform http://www.abysis.org/ (accessed on 30 August 2022). ScFv-encoding fragments with modified unusual residues were ordered as gene products (Atum, Newark, NJ, USA), and the original scFv coding region pCG-H-αBiotin was replaced after SfiI/NotI digestion. The GFP-encoding transfer plasmid pHR-SEW was described previously [15]. The transfer plasmid encoding a polycistronic expression cassette, containing the CD20-CAR followed by a P2A element and the transduction marker LNGFR, was generated by replacing the GFP-encoding region in pHR-SEW [10]. 

### 2.3. LV Generation, Concentration, and Titration

Third-generation LVs were generated via the transient transfection of HEK-293T cells as described previously [15,20]. Briefly, 4.5 × 10^6^ cells were seeded in T175 flasks 3 d prior to transfection with polyethyleneimine (PEI) and 35 μg of DNA mix. Both 48 and 72 h later, the supernatant was collected and filtered to remove cellular debris. The LV particles were concentrated via centrifugation through 20% sucrose (20% *w/v* in PBS, Sigma Aldrich, St. Louise, USA) for 24 h at 4 °C with 5350× *g*. The supernatant was decanted, and pellets were resuspended in PBS via thorough pipetting. Resuspended LVs were stored until further use at −80 °C. 

LV particles were quantified after one freeze–thaw cycle via titration on HT1080 cells (GFP) or SupT1 cells (CAR). One day prior to transduction, the cells were biotinylated using NHS-Biotin following the manufacturer’s instructions (Thermo Fisher, Waltham, MA, USA). Staining and flow cytometry was used to confirm biotinylation. Subsequently, HT1080 cells were seeded in complete medium with 1.1 × 10^5^ cells/well in 24-well plates and incubated overnight at 37 °C, 5% CO_2_. The SupT1 cells were cultivated overnight in complete medium and seeded in RPMI (2 mM stable glutamine) directly before transduction with 2 × 10^5^ cells/well in 96-well round-bottom plates. The cells were transduced with LV particles serially diluted in serum-free DMEM or RPMI (2 mM stable glutamine), respectively. Then, 3 h (HT1080) and 24 h (SupT1) post transduction, the complete medium was supplemented. The transduction efficiency was analyzed either 72 h (HT1080) or 96 h (SupT1) post transduction via flow cytometry. LV titers are represented as transducing units per volume (TU/mL), calculated by the ratio of transduced cells and applied LV volume [24].

### 2.4. Generation of Adapter Molecules

Adapter molecules were either commercially available (Miltenyi Biotec, Bergisch Gladbach, Germany) or were generated from IgG antibodies with the f(ab)_2_ or fab preparation kit following the manufacturer’s instructions (Thermo Fisher Scientific, Waltham, MA, USA). Generated f(ab)_2_s and fabs were labeled using NHS-Biotin (Thermo Fisher Scientific, Waltham, MA, USA) [24].

### 2.5. Flow Cytometry

The MACSQuant^®^ Analyzer 10 or the MACSQuant^®^ X (Miltenyi Biotec, Bergisch Gladbach, Germany) were used for flow cytometry analysis. The data were analyzed with the Flowlogic™ software (Inivai, Mentone Victoria, Australia) using either frequency or event count. Cells were stained in CliniMACS^®^ PBS/EDTA Buffer with 0.5% BSA (both Miltenyi Biotec, Bergisch Gladbach, Germany) at 4 °C for 10 min. Antibodies used for flow cytometry were obtained from Miltenyi Biotec and were used according to the manufacturer’s protocol.

### 2.6. Transduction of Cell Lines

Next, 2 × 10^5^ cells/well SupT1 cells were seeded in 96-well round-bottom plates, and 1 × 10^5^ cells/well Raji cells were seeded in 48-well plates in RPMI (2 mM L-glutamine). If not otherwise noted, the cells were incubated with adapter molecules in the indicated concentrations for 30 min at 4 °C. Subsequently, LVs diluted in RPMI with 2 mM glutamine were added, and the cells were incubated at 37 °C, 5% CO_2_. The complete medium was supplemented 3 h (Raji) or 24 h (SupT1) after transduction. Vectofusin^®^-1 (Miltenyi Biotec, Bergisch Gladbach, Germany) was used according to the manufacturer’s instructions. Polybrene^®^ was diluted with the LV to a final concentration of 8 μg/mL in the transduction volume. GFP-positive cells were measured via flow cytometry 72 h (Raji) and 96 h (SupT1) post transduction [24].

### 2.7. Transduction of PBMCs

PBMCs were seeded 1 d before transduction with 2.5 × 10^5^ cells/well in TexMACS™ medium (12.5 ng/mL IL-7, 12.5 ng/mL IL-15, 50 IU/mL IL-4 and TransAct™ (Miltenyi Biotec, Bergisch Gladbach, Germany)) in 96-well flat-bottom plates. The cells were incubated for 30 min at 4 °C with adapter molecules in the indicated concentrations, followed by transduction. If noted, Vectofusin^®^-1 was used following the manufacturer’s instructions. For spinoculation, the samples were centrifuged for 90 min at 1000 rpm, 32 °C. The cells were supplemented with 5% AB serum (Sigma Aldrich, St. Louise, USA) 1 d post transduction. The medium was replaced with fresh TexMACS™ (12.5 ng/mL IL-7, 12.5 ng/mL IL-15, 50 IU/mL IL-4, 1µg/mL CD40L) 2 d post transduction. GFP-positive cells were analyzed via flow cytometry 9 d post transduction [24].

### 2.8. Transduction of Murine T cells

Murine T cells were isolated from the spleens of C57BL/6 mice. To do so, a single-cell suspension from a mouse spleen was prepared using the program m_spleen_01.01 on the gentleMACS™ Dissociator (Miltenyi Biotec, Bergisch Gladbach, Germany). T cells were isolated from the single-cell suspension using the Pan T Cell Isolation Kit II (Miltenyi Biotec, Bergisch Gladbach, Germany) following the manufacturer’s instructions. The T cells were seeded in a density of 1.25 × 10^6^ T cells/mL in 96-well flat-bottom plates and activated overnight in TexMACS™ medium (30 IU/mL mIL2, Dynabeads Mouse T-activator CD3/CD28 in a bead to cell ratio of 1:1). The next day, T cells were preincubated with adapter molecules in concentrations of 500 ng/mL, as described previously. The T cells were transduced with a GFP-encoding Ad-LV and VSV-G LV with a dose of 5 TU/cell in the presence of Vectofusin^®^-1 for Ad-LVs only. GFP-positive cells were determined via flow cytometry 7 days post transduction by staining for CD3 (clone: REA641), CD4 (clone: REA1211), and CD8 (clone: REA601). 

### 2.9. Production of CAR-T cells

For the generation of CAR T cells, CD4+ and CD8+ T cells were negatively selected using the Pan T cell Isolation Kit (Miltenyi Biotec, Bergisch Gladbach, Germany). The cells were seeded in a density of 1 × 10^6^ T cells/mL in 96-well flat-bottom plates and activated overnight in TexMACS™ (TransAct™,12.5 ng/mL IL-7, 12.5 ng/mL IL-15). Next, the activated T cells were incubated with adapter molecules as described previously (CD4: 100 ng/mL, CD8: 100 ng/mL, CD4/CD8 (1:1): 50 ng/mL each, CD4/CD8 (5:1) 80 ng/mL CD4 and 20 ng/mL CD8 f(ab)_2_). Ad-LVs encoding a 2nd-generation CD20-CAR comprising a Leu16-derived scFv, CD8 hinge, CD8 transmembrane domain, 4-1BB-costimulatory domain, and CD3 zeta stimulatory domain were used for transduction at a dose of 0.5 TU/cell [10]. Vectofusin^®^-1 was used as described in the manufacturer’s instructions. Then, 48 h post transduction, the medium was replaced with fresh TexMACS™ (12.5 ng/mL IL-7, 12.5 ng/mL IL-15). The T cells were splitted every other day. Phenotype and transduction efficiency were evaluated 5 and 12 days post transduction. 

### 2.10. Cytotoxicity Assay and Cytokine Quantification

For the evaluation of cytolytic capacity, CAR-T cells were co-cultured with GFP+ Raji cells at an E:T ratio of 1:1 for 6 days. Following the manufacturer’s instructions, the supernatant was analyzed for secreted cytokines after 24 h of the co-culture using the MACSPlex Assay (Miltenyi Biotec, Bergisch Gladbach, Germany). Residual tumor cells were counted after 24 h using flow cytometry. Additionally, T cells were analyzed for the expression of exhaustion markers after 6 days of co-culture using flow cytometry.

## 3. Results

### 3.1. Adapter-Mediated Lentiviral Vectors Are Flexible towards Antigen Specificity

**Figure 1:**
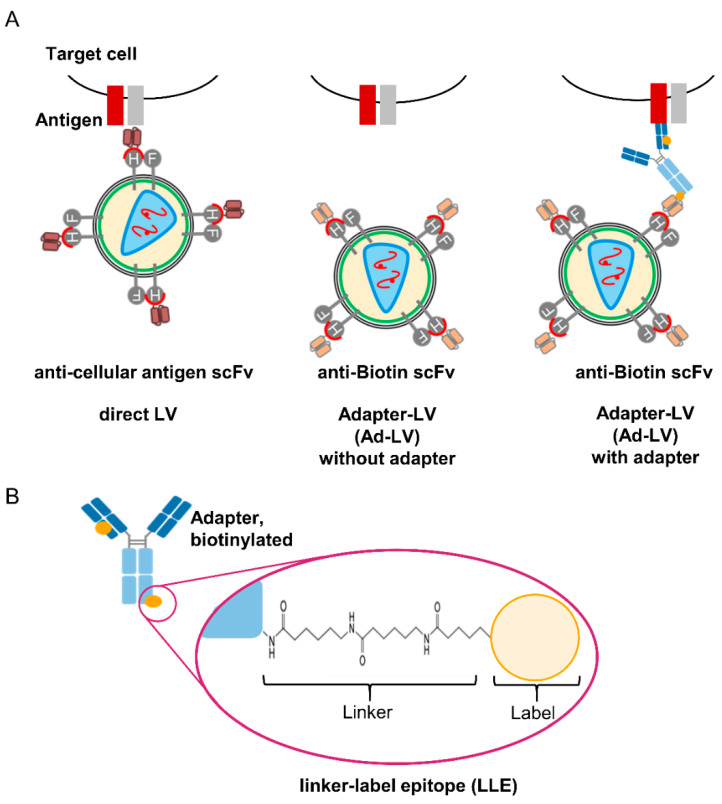
Concept of LLE adapter-mediated transduction with lentiviral vectors. (**A**) For directly targeted LVs, a binding ligand (e.g., scFv) is fused to the H protein of MV that is mutated to block natural receptor interactions. This allows specific entry to cells expressing the respective antigen (left). The adapter-mediated transduction is based on an scFv fused to the H protein of MV pseudotyped LVs, which binds to biotin on a specific adapter molecule. In the absence of adapter molecules, transduction is abolished (middle). Only in the presence of a biotinylated adapter molecule transduction with the Adapter-LV (Ad-LV) is mediated (right). (**B**) Here, antibodies or antibody fragments were used as adapter molecules. The specific linker chemistry used for biotinylation generates a linker moiety and a label moiety (biotin). The scFv used in this study interacts with the linked biotin (linker–label epitope, LLE) thereby avoiding recognition of free biotin.

LVs pseudotyped with measles virus glycoproteins are a well-established platform for the targeted transduction of cells [5]. For retargeting, the H glycoprotein is mutated to ablate binding to the natural receptors [25]. Binding is restored by C-terminal fusion of an scFv that is specific for the antigen of choice on the target cell, e.g., CD20, CD105, or CD8 [15,17,26]. Consequently, only cells expressing the respective antigen are transduced. To generate flexible targeted LVs, a biotin-specific scFv (mBio3-18E7) that only binds tagged adapter molecules is fused to the H protein (Figure 1A) [23]. 

Only in the presence of a biotinylated adapter molecule Ad-LV particles bind and transduce the target cell. The target specificity is easily exchanged by variation in the adapter specificity, without the need of additional protein engineering. The linker chemistry used for biotinylation results in adapter molecules that are composed of a linker moiety and a label moiety (biotin). The mBio3-18E7 scFv binds biotin in the context of the specific linker, referred to as the linker–label epitope (LLE) (Figure 1B) [23]. Consequently, unspecific binding to free or cell-bound biotin is avoided.

**Figure 2:**
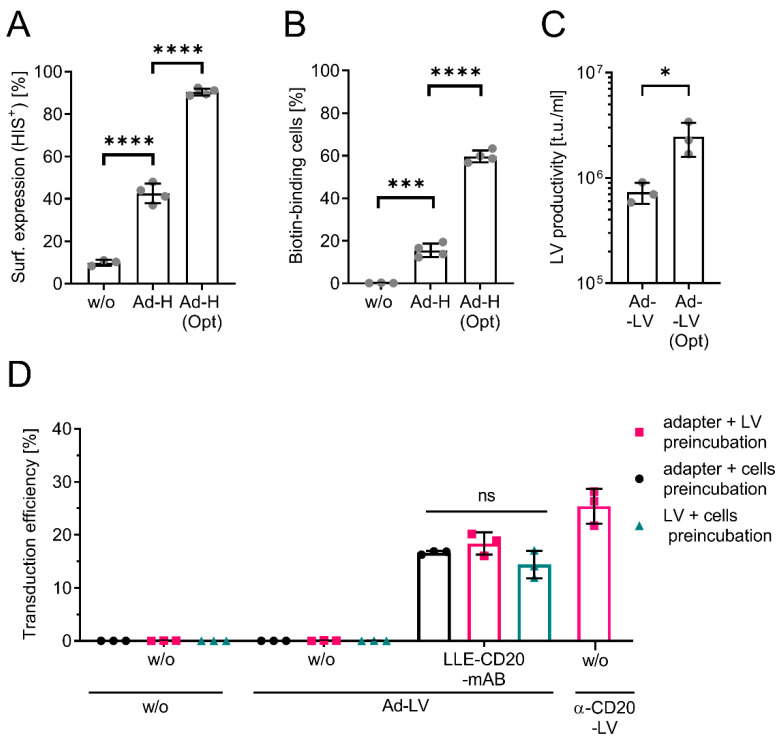
Establishing the Ad-LV system. (**A**,**B**) Flow cytometric analysis of the surface expression of the H protein fused to the original scFv or a mutant variant (Opt) on HEK293T cells by either staining for the C-terminal HIS-tag (**A**) of the H protein or with fluorescently labeled biotin (**B**) or untreated cells (w/o). Data are represented as mean ± SD of 4 independent experiments. (**C**) LV productivity for concentrated LV supernatant was determined on biotinylated cells. Data are represented as mean ± SD of 3 independent experiments. (**D**) The optimal order of combination of the three components (LVs, cells, and adapter) required for transduction was evaluated via transduction of Raji cells with a GFP-encoding Ad-LV (0.05 TU/cell) using an LLE-CD20-mAB (clone: LT20, 1000 ng/mL). While cells with vector were preincubated for 30 min at 37 °C, cells with adapter or vector with adapter were preincubated for 30 min at 4 °C. Afterwards, the respective third component was added. Transduction without the addition of any adapter (w/o) was used to confirm specificity. MV-LV directly targeting CD20 (α-CD20-LV) was used as positive control. Transduction efficiency was analyzed 3 days post transduction via quantification of the GFP-positive cells using flow cytometry. Data are represented as mean ± SD of 3 technical replicates. Statistic ordinary one-way ANOVA using Tukey’s multiple comparisons test; ns, non-significant; * *p* < 0.05, *** *p* < 0.001, **** *p* < 0.0001.

The efficiency of pseudotyping correlates with the surface expression of the H protein in the producer cells [27]. Since initial experiments revealed a low surface expression of the designed protein, one critical residue was identified and converted to the murine germline consensus sequence, resulting in Ad-H(opt) [27]. Surface expression was analyzed in the producer cells HEK293T transfected with the plasmid encoding the H protein fused to the original (Ad-H) or the optimized mBio3-18E7-derived scFv. Expression levels were quantified by staining either for the C-terminal HIS-tag of the H protein (Figure 2A) or with fluorescently labeled biotin as the antigen of the scFv and subsequent flow cytometric analysis (Figure 2B). 

A 2-fold increase in HIS-positive cells and a 4-fold increase in biotin-binding cells was observed when Ad-H(opt) was expressed, suggesting improved surface-expression and antigen-binding capacity. Next, LVs were generated via the transient transfection of HEK293T cells. To determine an adapter-independent functional titer, LVs were titrated on biotinylated cells. For this, surface proteins of HT1080 cells were randomly labeled using NHS-linked biotin, followed by transduction with serially diluted LVs and the quantification of GFP-expressing cells via flow cytometry (Figure S1). In line with improved surface expression, the productivity of the Ad-LV generated with the Ad-H(opt) was enhanced 3-fold as compared to Ad-H (Figure 2C and Figure S1). The significant increase in productivity prompted us to apply Ad-LVs with Ad-H(opt) in all subsequent experiments. 

Having shown that Ad-LVs can be produced at sufficient scales to run in vitro experiments, we next evaluated adapter-mediated transduction by testing CD20 as our first target for vector binding. For this, the three components, cells, adapter, and LVs, must be combined in the most optimal order. We evaluated the preincubation of two components for 30 min followed by the addition of the respective third. For this, CD20-expressing Raji cells were transduced with a GFP-encoding Ad-LV in the absence or presence of an LLE-conjugated CD20 mAB (LLE-CD20 mAB). A directly targeted α-CD20-LV was used as reference [15]. The transduction efficiency was measured 3 days later through the analysis of GFP-positive cells using flow cytometry. Raji cells were transduced in the presence of LLE-CD20 mAB only, while no GFP-positive cells were detected in the absence of an adapter (Figure 2D). The transduction efficiency was not influenced by the order of combining the components. Considering the differences in functional titer analysis (titration on biotinylated HT1080 vs. titration on Raji cells), the transduction efficiency with Ad-LVs and α-CD20-LVs was comparable. All subsequent transductions were performed via the preincubation of cells with adapter molecules for 30 min followed by the addition of the LVs. 

When applying gammaretro- and lentiviral vectors in vitro, the transduction efficiency can be increased by applying transduction enhancers [28]. Hence, we assessed whether the transduction rates of Ad-LVs can be improved using Polybrene^®^ or Vectofusin^®^-1. While Polybrene^®^ improved the transduction efficiency only by 1.5-fold, a 4-fold increase in transduction was achieved using Vectofusin^®^-1 (Figure S2). This is in line with published data on retroviral vector systems [20,29]. 

**Figure 3:**
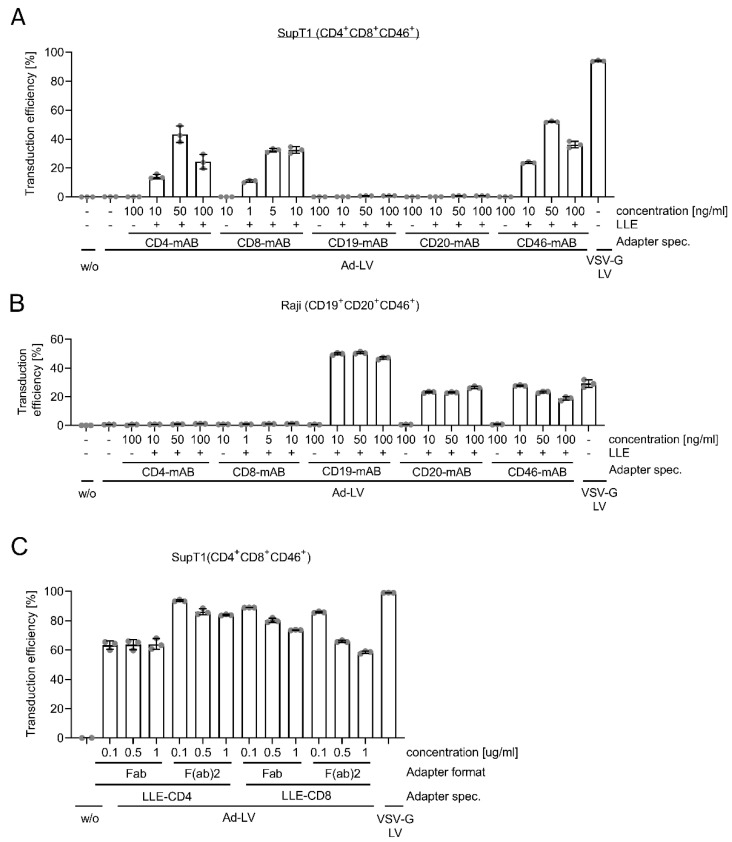
Flexibility of Ad-LV towards adapter specificity and format. The flexibility of the adapter system was evaluated by transduction of cell lines using different adapter specificities. SupT1 (CD4 + CD8 + CD46+) (**A**) and Raji cells (CD19 + CD20 + CD46+) (**B**) were left untreated (w/o) or were transduced with GFP-encoding Ad-LV with 0.2 TU/cell using LLE-CD4 (clone: MT-466), LLE-CD8 (clone: BW135/80), LLE-CD19 (clone: LT19), LLE-CD20 (clone: LT20), and LLE-CD46 (clone: REA312) mABs (+) in concentrations ranging from 1 to 1000 ng/mL. Non-biotinylated mABs (-) used in the highest concentration and transduction in the absence of any adapter (-) were used to confirm specificity. VSV-G LV was used as reference. Transduction efficiency was analyzed 3 days (Raji) or 4 days (SupT1) post transduction via quantification of GFP-positive cells using flow cytometry. (**C**) Transduction efficiency of SupT1 comparing fab and f(ab)_2_ adapter formats of the same LLE-CD4 or LLE-CD8 clone with adapter concentrations ranging from 0.1 to 1 μg/mL using a GFP-encoding Ad-LV (0.1 TU/cell). VSV-G LV was used as reference. Data are represented as mean ± SD of 3 technical replicates.

Next, alternative adapter specificities were applied to investigate whether Ad-LV is a generic system, being able to transduce alternative cell types as well. Two cell lines, SupT1 and Raji cells, which vary in the expression of surface markers, were transduced with GFP-encoding Ad-LVs in the presence of LLE-conjugated or non-biotinylated mABs specific for CD4, CD8, CD19, CD20, and CD46 (Figure 3A,B). Three different concentrations of the antibodies were used as the antibody affinity and antigen expression level could influence the amount needed. VSV-G pseudotyped LVs were used as the reference. Again, transduction levels were analyzed via the quantification of the GFP-expressing cells using flow cytometry. Both cell lines were transduced in the presence of LLE-conjugated mABs only when the respective antigen was expressed. No transduction was observed when non-biotinylated mABs or no adapter were added. The expression levels of GFP did not differ over a period of 21 days, excluding pseudotransduction and confirming LV-mediated gene transfer (Figure S3). Of note, the optimal adapter concentration varied between adapter specificities. CD4-mediated transduction efficiency levels peaked at 44% with 50 ng/mL of the adapter. In contrast, CD19-specific transduction ranged at around 50% irrespective of the antibody concentration applied. However, declining transduction levels are easily conceivable in the presence of even higher antibody concentrations. At high adapter concentrations when threshold levels are reached, the adapter is completely bound to Ad-H(opt) on the surface of the Ad-LV, and at the same time, in a saturating manner to the cellular antigen expressed by the target cell. Thus, when the adapter is provided in excess, the Ad-LV may not bind anymore to the cellular antigen, resulting in lower transduction levels, as seen in the presence of 100 ng/mL LLE-CD4 mAB or LLE-CD46 mAB on SupT1. The onset of the saturation effect is dependent on antigen expression level and antibody affinity, explaining the observed differences. Consequently, the titration of adapter molecules is recommended. 

While SupT1 cells were most efficiently transduced with VSV-G LVs, Ad-LVs in the presence of an LLE-CD19 mAB induced the highest transduction efficiency on Raji cells. Low LDLR expression may have limited VSV-G LV binding and subsequent transduction. 

The targeted epitope and the adapter format may have an influence on the distance of Ad-LVs and the cellular membrane. For alternative pseudotypes, the distance was shown to be important for fusion [14]. The adapter influences this distance by the location of the epitope on the antigen but also by its size. Hence, smaller antibody fragments, e.g., fab and f(ab)_2_, were evaluated to serve as adapter molecules. For this, SupT1 cells were transduced with GFP-encoding Ad-LVs in the presence of LLE-CD4 and LLE-CD8 fab or f(ab)_2_ molecules in increasing concentrations (Figure 3C). For CD8, no significant difference in transduction efficiency was observed for both adapter formats. In contrast, LLE-CD4 f(ab)_2_ molecules outperformed LLE-CD4 fabs, suggesting that in addition to affinity, the adapter avidity may influence the transduction. For all adapter molecules except LLE-CD4 fabs, transduction was reduced with an increasing concentration, suggesting a saturation effect, as described above. 

### 3.2. Ad-LV Selectively Transduces Target Cells in Mixed Cell Populations 

Next, we quantified the selectivity of Ad-LVs in mixed cell populations of two cell lines. The influence of several transduction enhancers was evaluated as well to investigate potential effects on the selectivity. Co-cultured Raji and cell-trace-dye-labeled SupT1 cells were transduced with GFP-encoding LVs in the presence of LLE-CD4, LLE-CD8, and LLE-CD20 mABs. 

**Figure 4:**
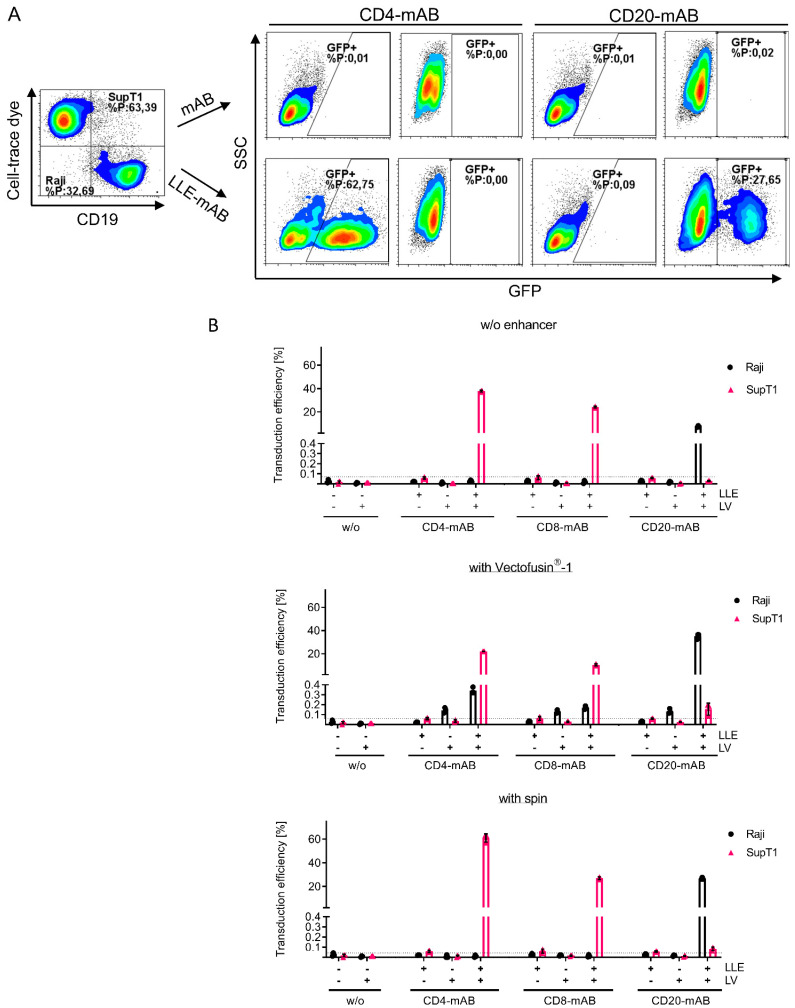
Selectivity of Ad-LVs in co-culture assay using different transduction protocols. To confirm selectivity of Ad-LVs, target cells were transduced with 0.2 TU/cell in a co-culture with non-target cells. Cell-trace-dye-labeled SupT1 cells (CD4+, CD8+, and CD46+) were co-cultivated in a 1:1 ratio with Raji cells (CD19, CD20+). (**A**) Representative data are shown using spinoculation. The co-culture was left untreated (-) or was transduced with Ad-LVs in the presence of non-biotinylated mAB (-) or LLE-CD4 mAB (clone: MT-466, 50 ng/mL), LLE-CD8 mAB (clone: BW135/80, 10 ng/mL) or LLE-CD20 mAB (clone: LT20, 500 ng/mL). (**B**) Three transduction conditions were compared using no enhancer, using the transduction enhancer Vectofusin^®^-1, or using spinoculation. Transduction efficiency was evaluated 4 days post transduction via gating on the cell-trace-dye-labeled SupT1 and the CD19-expressing Raji cells. Data are represented as mean ± SD of 3 technical replicates.

As a control, antibodies without biotinylation were used. Centrifugal inoculation, or spinoculation, was used as a physical transduction enhancer and compared to the chemical enhancer Vectofusin^®^-1. GFP-expressing cells were quantified on each population separately by gating on cell-trace-dye-labeled SupT1 and CD19+ Raji cells (Figure 4A). 

In the absence of any enhancer, or when spinoculation was applied, the off-target transduction was below the detection limit independent of the antibody clone (Figure 4B). In addition, no detectable transduction was measured in the absence of an adapter or in the presence of a non-biotinylated adapter. Interestingly, spinoculation only enhanced CD4- and CD20-specific transduction. In accordance with our previous data, Vectofusin^®^-1 augmented the transduction efficiency on Raji cells by 4-fold. In contrast, the transduction of SupT1 cells was reduced after the complexation of Ad-LV particles with Vectofusin^®^-1. Off-target effects close to the detection limit (up to 0.3% GFP-positive CD4/8-negative Raji cells, up to 0.2% on CD20-negative SupT1 cells) in the presence of Vectofusin^®^-1 were observed, indicating that the ligand specificity of Ad-LVs was maintained. Depending on the adapter specificity and the transduction enhancer used, on-target to off-target ratios ranged from 70- to 5000-fold.

### 3.3. Ad-LV Selectively Transduces Activated Primary Human and Murine Lymphocytes 

**Figure 5:**
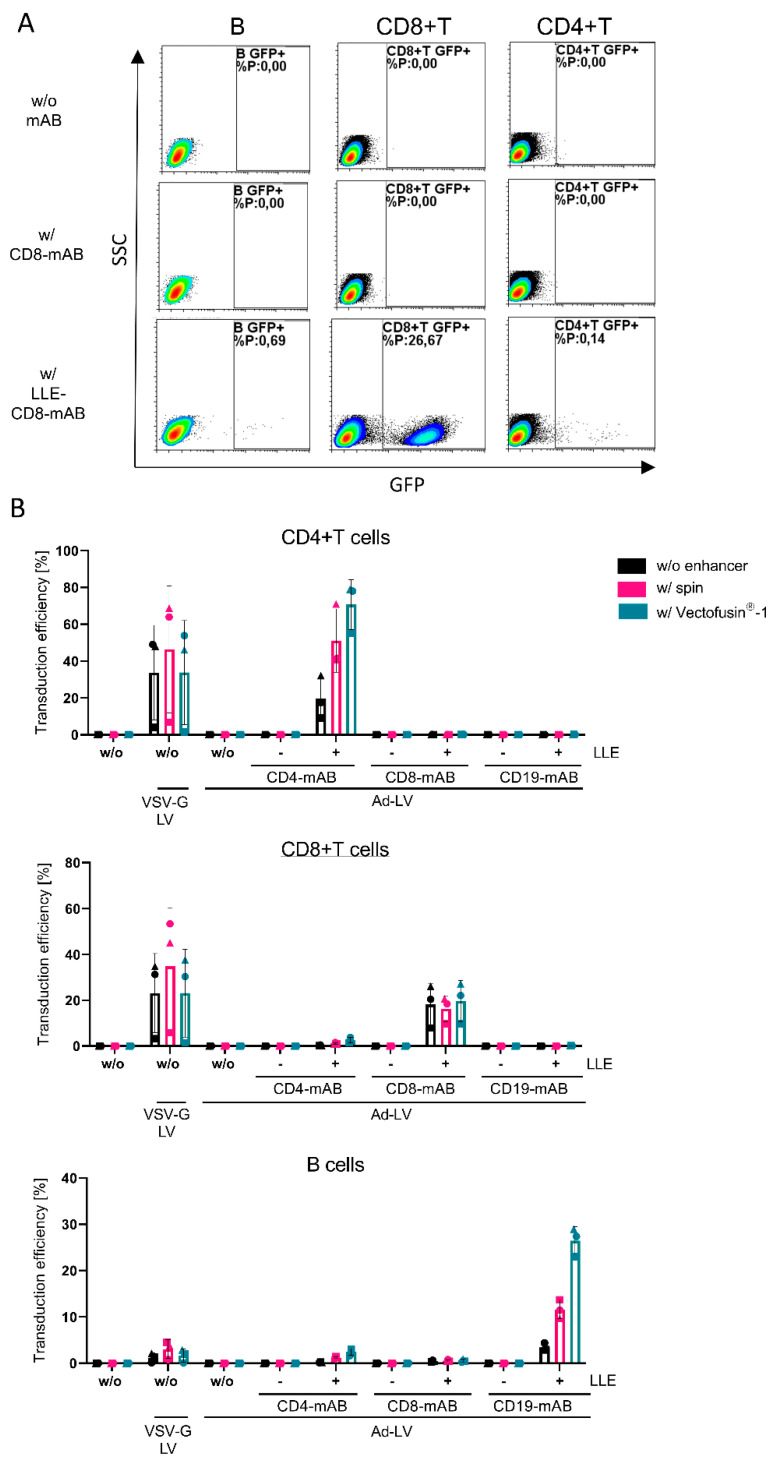
Selective transduction of human T and B cells within activated PBMCs using different transduction protocols. Activated PBMCs were transduced with Ad-LVs with 0.1 TU/cell in the absence of adapter (w/o) and in the presence of CD4-mAB (clone:MT-466, 100 ng/mL), CD8-mAB (clone: BW135/80, 10 ng/mL), or CD19-mAB (clone: LT19, 100 ng/mL) with (+) or without LLE-tag (-). (**A**) Representative data are shown using LLE-CD8 mAB or CD8-mAB w/o enhancer. (**B**) Three transduction conditions were compared: using no enhancers, using spinoculation, or the transduction enhancer Vectofusin^®^-1. VSV-G LVs were used as positive control. Transduction efficiency was evaluated 10 days post transduction gated on the subpopulations (CD4+ T cells, CD8+ T cells, and B cells). Data are represented as mean ± SD of 3 different donors and 2 independent experiments.

To assess the selectivity on primary cells, we established a transduction and cultivation protocol, which allows the simultaneous transduction of primary human T and B cells directly within PBMCs without enrichment. Freshly isolated PBMCs were cultivated in TexMACS™ medium supplemented with IL-7, IL-15, and IL-4. For the activation of T and B cells, we added TransAct™ and CD40L, respectively. PBMCs were transduced one day later with GFP-encoding Ad-LVs in the presence of LLE-CD4, LLE-CD8, and LLE-CD19 mAB. Transduction in the presence of non-biotinylated mAB was used as the control. Again, different transduction enhancers were compared, and a non-selective VSV-G pseudotyped LV served as the control. The transduction efficiency was analyzed on different cell populations, namely CD4+ T cells (CD19-, CD3+, CD56-, and CD4+), CD8+ T cells (CD19-, CD3+, CD56-, and CD4+), and B cells (CD19+) (Figure 5A).

In contrast to the selectivity assay on cell lines, the discrimination of the different populations within PBMCs via flow cytometry was more challenging. Again, no transduction was detected when a non-biotinylated adapter or no adapter was added, confirming the previous results. As for cell lines, transduction levels in the presence of LLE-CD8 mAB were not increased by any transduction enhancer, while CD4- and CD19-specific transduction levels were higher for both. As expected, on B cells, Ad-LVs outperformed VSV-G LVs, as they are known to be refractory to VSV-G-mediated transduction due to low LDLR expression even in an activated status [8]. Depending on the adapter specificity and the transduction enhancer used, on-target to off-target ratios of about 50–250-fold were observed (Figure 5B).

**Figure 6:**
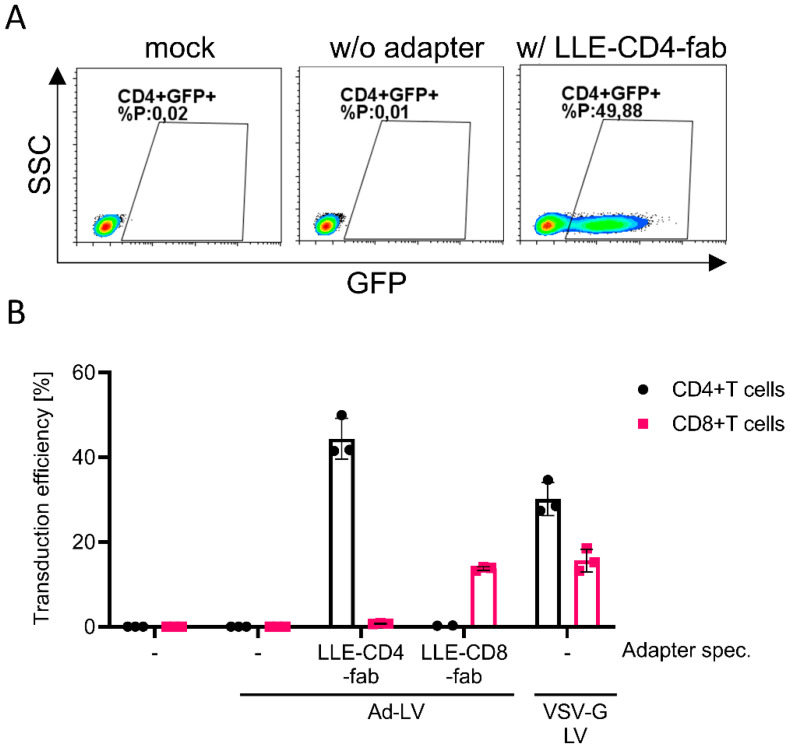
Selective transduction of activated murine T cells. Murine T cells were isolated from murine spleens, activated overnight, and transduced using Ad-LVs at a dose of 5 TU/cell. (**A**) Representative data are shown of CD4+ T cells transduced in the absence of adapter or in the presence of LLE-CD4 fab. (**B**) Isolated murine T cells from spleen were either left untreated (-) or transduced with Ad-LVs in the absence of adapter (-) or in the presence of LLE-CD4 fab (clone: GK1.5) and LLE-CD8 fab (clone: 53–6.7)-specific adapter molecules (500 ng/mL). VSV-G LVs were used as positive controls. Data are represented as mean ± SD of 3 different mice.

Next, we evaluated if Ad-LVs may also be used for the selective transduction of murine T-cell subsets. Murine T cells were isolated from the spleens of C57BL/6 mice and stimulated overnight with IL-2 and mouse T-activator. The cells were either left untreated or were transduced with Ad-LVs in the absence of an adapter or in presence of LLE-CD4 and LLE-CD8 fab (Figure 6). 

VSV-G LVs were used as controls. The transduction efficiency was analyzed 7 days later via the quantification of GFP-expressing cells using flow cytometry. In the absence of an adapter, transduction efficiency levels below the detection limit were measured, while in the presence of an adapter, only the respective target population was transduced. As expected, VSV-G LVs transduced both populations. CD4+ T cells were transduced with higher efficiency than CD8+ T cells with both VSV-G LVs (2-fold) and Ad-LVs (3-fold). Of note, to achieve similar transduction efficiency levels on murine T cells as compared to human T cells, a 25-fold higher LV dose was required.

### 3.4. Adapter Mixing allows Generation of CAR-T Cells with Varying CD4/CD8 Ratios from Activated Pan T cells 

Having shown that Ad-LVs can be used for the genetic modification of T cells, we aimed to explore their application in the field of CAR-T cell therapy. Targeted transduction can be employed in multiple ways, e.g., as a tool for the analysis of CAR-T functionality or for the manufacturing of CAR-T cells. 

The manufacturing process for CAR-T cells is being constantly refined to increase the therapeutic efficacy of CAR-T cells as well as to reduce the complexity of the process and thereby production costs. Both the T-cell phenotype and T-cell subset in the starting material and final product were shown to influence the therapeutic efficacy of CAR-T cells. For instance, the application of defined ratios of T-cell subsets or manufacturing from naïve/stem memory T cells was shown to enhance anti-tumor reactivity and CAR-T persistence [12,30,31]. Hence, a better understanding of the synergistic effects of different T-cell subsets within the CAR-T product will help to improve the therapeutic efficacy. To do so, state-of-the-art methods require the isolation of subpopulations, separate genetic modification, and the subsequent mixing of the cell products [12,30,31,32]. In contrast, targeted LVs can be used to transduce defined T-cell subsets within mixed populations. For instance, Agarwal et al. generated CAR-T cells with varying CD4/CD8 ratios directly from PBMCs by the mixing of LVs [19]. 

Here, we used Ad-LVs to analyze the function of CD4+ and CD8+ CAR T cells in a hematological in vitro tumor model. For this, CD20-CAR-T cells with varying CD4/CD8 ratios were generated in a single transduction step from activated Pan T cells simply via mixing of the respective adapter molecules. This way, all samples are treated with the same LV batch and cultivated under the exact same conditions, enabling us to study the function of the different subsets.

**Figure 7:**
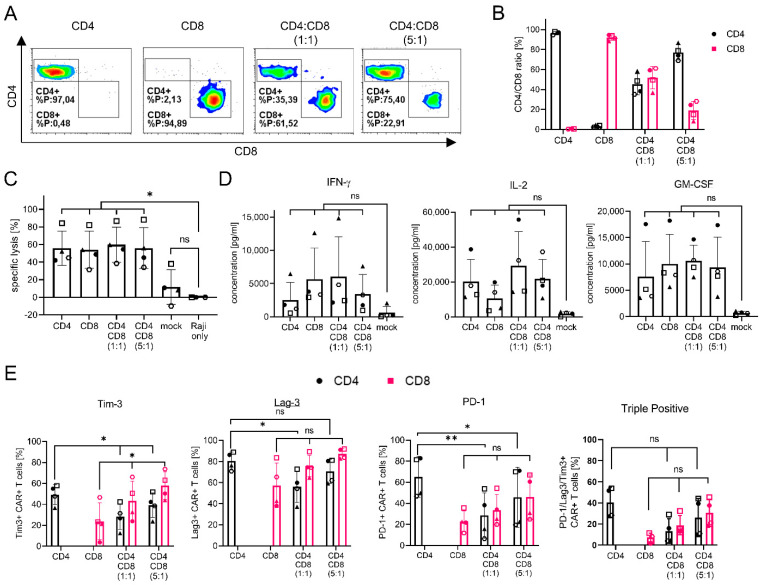
Generation of CD20-CAR-T cells with Ad-LVs. CAR-T cells were generated from activated Pan T cells using Ad-LV (0.5 TU/cell). To generate CAR-T cells with varying CD4/CD8 ratios, either LLE-CD4 f(ab)_2_ or LLE-CD8 f(ab)_2_ adapters alone or a mixture of both adapters in 1:1 or 1:5 ratio was used. (**A**) Representative data of T cells transduced with Ad-LVs using LLE-CD4 f(ab)_2_ (clone: MT-466) or LLE-CD8 f(ab)_2_ (clone: BW135/80) alone or in a mixture are shown. (**B**) The CD4/CD8 ratio of the CAR-T cells is shown. To analyze if the generated CAR-T cells are functional co-cultures with CD20, + GFP+ Raji cells were set up in an E:T ratio of 1:1. (**C**) Specific lysis of target cells was analyzed via flow cytometry 24 h post setup of the co-culture. (**D**) Secretion of specific cytokines was analyzed using the MACS Plex Assay Kit. (**E**) Activation/exhaustion marker expression was analyzed 6 days post setup of the co-culture assay. Data are represented as mean ± SD of 4 different donors. Statistics: repeated measure one-way ANOVA using Dunnett’s or Tukey’s multiple comparison test; ns = non-significant * *p* < 0.05, ** *p* < 0.01.

Activated Pan T cells were transduced with a CD20-CAR encoding Ad-LV in the presence of an LLE-CD4 or an LLE-CD8 f(ab)_2_, either alone or in a mixture. The transduction efficiency was analyzed via staining of the co-expressed transduction marker LNGFR using flow cytometry 12 days post transduction (Figure 7A,B).

As expected, in the presence of only one adapter, either only CD4+ or CD8+ CAR-T cells were generated. In the presence of a mixture of the LLE-CD4 and LLE-CD8 f(ab)_2_, both subsets were transduced. The CD4/CD8 ratio of the CAR-T product correlated with the ratio of the adapters present during transduction. Of note, the transduction efficiency levels were comparable (Figure S4). 

To investigate the role of CD4+ and CD8+ T-cell subsets in anti-tumor activity, we evaluated the specific lysis capacity of the CAR-T cells via co-cultivation with GFP + CD20+ Raji cells in an effector to target ratio (E:T) of 1:1. The lysis of tumor cells was analyzed via quantification of the fraction of GFP-expressing Raji cells 24 h after the setup of the co-culture using flow cytometry (Figure 7C). On average, 55% target cell lysis was observed in the presence of CAR-T cells, while no significant decrease in tumor cells was observed upon treatment with unmodified T cells. In line with published data, cytotoxicity against the tumor cells was independent of the CD4/CD8 ratio of the CAR-T cells, confirming the equal cytolytic activity of CD4+ and CD8+ T cells [33]. The quantification of the cytokine release confirmed the lysis data. Cytokine secretion was only observed upon co-cultivation with CAR-T cells and not with non-modified T cells (Figure 7D). 

Cytokine levels were strongly donor-dependent, resulting in no significant differences between the CAR-T products. Nevertheless, a tendency toward the enhanced secretion of IFN-γ was observed with higher frequencies of CD8+ CAR-T cells, while IL-2 secretion dropped in the absence of CD4+ CAR-T cells. Finally, 6 days post setup of the co-culture, the exhaustion/activation surface markers of the T cells were analyzed (Figure 7E). The ratio of CD4+ CAR-T cells expressing Tim-3 or PD-1 significantly dropped in the presence of CD8+ CAR-T cells. In contrast, Tim-3 expression on CD8+ CAR-T cells significantly increased in the presence of CD4+ CAR-T cells. No significant differences were observed in Lag-3 for CD8+ CAR-T cells, while expression on CD4+ CAR-T cells was only reduced with a high amount of CD8+ CAR-T cells. The same tendencies were observed on triple-positive T cells; however, no significant difference was observed. These effects are results of the fact that transduction can be excluded, since no significant differences in marker expression were observed before the co-culture setup (Figure S4). Most importantly, no significant differences in marker expression were observed on the non-modified T cells, confirming a CAR-specific effect (Figure S4). The additional analysis of less favorable E:T ratios, tumor re-challenge, and the analysis of phenotype and gene expression may uncover further differences between CD4+ and CD8+ CAR-T cells. Since the result will likely be affected by CAR design, the binding domain, and tumor entities, the evaluation of anti-tumor activity with additional tumor cell lines as well as in vivo analysis would be of value. 

In summary, Ad-LVs may be used to easily study the function of T-cell subsets in complex mixtures for immunotherapy, including a wider range of CD4/CD8 ratios. A study in the context of specific CARs of interest could help to identify superior CD4/CD8 ratios. Furthermore, Ad-LV technology now enables the study of each subset in more detail. 

## 4. Discussion

The widespread application of retargeted LVs is hampered by the time-consuming LV engineering required when developing novel applications and alternative specificities. Here, we report the development of a versatile targeting system for LVs that solely requires the addition of a biotinylated adapter molecule to enable highly selective gene transfer to various cell types. Compatibility with a wide range of readily available biotinylated molecules provides an ideal technology for a variety of applications. 

The reasons to choose LVs pseudoptyped with MV glycoproteins as a basis for the versatile targeting system are threefold. First, they provide highly selective transduction with off-target transduction levels below the detection limit in vitro and in vivo [15,18,19]. Second, successful retargeting to a broad range of antigens (e.g., CD20, CD19, CD30, CD4, CD8, CD133, and CD105) suggested the required range of flexibility [15,17,18,26,27]. Third, the successful transduction of primary immune cells, e.g., T and B cells even in quiescent state, is promising for applications in the field of immunotherapy [21,22]. 

Since no LV engineering is required, Ad-LVs enable faster screening for possible target receptors compared to retargeted MV-LVs. Efforts to retarget MV-LVs to new receptors generally result in active receptor-targeted LVs only approximately 50% of the time [5]. In contrast, Ad-LVs are not dependent on the expression of a binding ligand in the context of the H protein and will thereby increase the number of possible targets. As only one LV batch needs to be produced to transduce different cell types, screening processes will be more efficient. In addition, safety is increased by rendering the LVs non-functional in the absence of LLE-conjugated adapters. Moreover, transduction efficiency is simply regulated by adapter concentrations, and the duration of the transduction reaction can be adjusted as needed by choosing adapters with short or long half-lives. 

Multiple strategies have been developed to render retargeted lentiviral vectors versatile by the addition of adapter molecules or antibodies. Sindbis pseudotyped LVs have been retargeted by the integration of biotin adaptor proteins, which bears the risk of unspecific adsorption to non-biotinylated proteins [34]. Moreover, avidin and streptavidin may bind to free biotin or cell-bound biotin, resulting in unspecific transduction. To ensure selectivity, we chose an scFv specific for biotin in the context of an LC-LC linker to reduce unspecific binding to free biotin or protein-bound biotin. Versatile targeting systems based on split-inteins, protein–peptide pairs, or bispecific antibodies have been developed, which require protein engineering for every new adapter specificity [35,36,37,38]. For Ad-LVs, adapter molecules of various specificities are commercially available and may otherwise be readily generated by the coupling of biotin to the desired molecule. 

Productivity is of major importance to enable broad application in the field of basic research and gene therapy. Unconcentrated VSV-G LVs are produced in ranges from 1 to 5 × 10^7^ TU/mL, approximately 50–100-fold higher than functional titers of Ad-LVs (Figure 2C). This is in accordance with published data of the targeted MV-LVs but also shows a limitation of the designed system [15,20]. Boosting transduction efficiencies by using transduction enhancers such as spinoculation and Vectofusin^®^-1 can partially compensate for low productivity. Optimizing the manufacturing protocol offers the potential to increase yields. Process optimization for Ad-LVs is much more efficient, as optimization steps are not needed for each individual specificity. The additional sequence optimization of the mBio3 scFv may yield improved surface expression and in turn higher productivity. Importantly, Ad-LVs may be used at lower doses compared to VSV-G LVs on mixed populations, as there is no loss of vector particles to off-target cells. 

The selectivity of Ad-LVs was initially assessed via the transduction of co-cultured target and non-target cells lines (Figure 4). In the absence of an enhancer, off-target transduction efficiency levels were below the detection limit irrespective of whether no adapters, non-biotinylated adapters, or non-specific LLE-conjugated adapters were present. Moreover, spinoculation enhanced transduction efficiency but did not affect the selectivity. In contrast, the presence of Vectofusin^®^-1 induced minimal off-target transduction at maximal frequencies of 0.399%. Interestingly, off-target transduction induced by Vectofusin^®^-1 was higher in the presence of LLE-conjugated antibodies as compared to off-target transduction in the absence of adapters. This suggests that Vectofusin^®^-1 may stabilize non-specific antibody interactions. In summary, the highest on-to-off target transduction ratios were observed upon the application of spinoculation (300–5000-fold), followed by transduction without enhancers (260–1300-fold) and transduction in the presence of Vectofusin^®^-1 (60–280-fold). Comparable results were obtained for the evaluation of the off-target transduction of PBMCs (Figure 5). However, on-target to off-target transduction ratios of only 30–280-fold were observed when LLE-conjugated adapters were added. This discrepancy may be a result of the more complex co-culture and separation of the different cell types via flow cytometry. Ad-LVs provide a higher level of selectivity compared to versatile targeting systems based on Sindbis pseudotyped LVs (on-to-off target ratios of 10–50-fold on cell lines and 25–115-fold on primary cells) [35,36]. In addition, retargeted GFP-encoding Sindbis-LVs transduced co-cultured cell lines above background levels even in the absence of adapters, while with Ad-LVs, the transduction efficiencies were below the detection limit [34]. Alphaviruses such as Sindbis-LVs enter cells via endocytosis, whereas paramyxoviruses fuse at the cell membrane, pointing towards the importance of alternative entry pathways for the selectivity. 

The selective transduction of target cells in complex cell compositions represents a major goal, especially for cell and gene therapy approaches. For instance, non-selective systems in vivo would result in low transduction efficiency levels on the target cell type of interest and bear the risk of toxicities by delivery to off-target cells. However, this has been seen also ex vivo, leading to adverse events, e.g., the off-target transduction of malignant B cells resulted in the resistance of the tumor cells to the CAR-T cells [13]. Hence, Ad-LVs have the potential to improve the safety of cellular therapies. 

Cell lines as well as human and murine primary cells were transduced using Ad-LVs (Figure 4,Figure 5 and Figure 6). In total, seven different antigens were targeted (CD4, CD8, CD19, CD20, CD46, mCD4, and mCD8), and three adapter formats were successfully used, demonstrating the flexibility and broad applicability of Ad-LVs. The adapter size, avidity, and half-life can be adjusted to the targeted antigen and experimental needs. Additionally, natural ligands such as interleukins or growth factors may be considered as adapter molecules [34,39]. The Ad-LV technology may be employed by scientists in clinical and basic research to transduce cells lines or primary cells that are refractory to state-of-the-art LVs, e.g., VSV-G pseudotyped LVs. 

Finally, we used Ad-LVs to generate CD20-CAR-T cells with varying CD4/CD8-ratios in a single transduction step simply via the mixing of adapter molecules (Figure 7). To the best of our knowledge, this is the first data showing the application of a versatile targeting system for a combinatorial approach. To date, two main strategies are used to analyze the functional differences between T-cell subsets: the transduction of bulk populations followed by single-cell RNA sequencing or the transduction of presorted T-cell subsets [32,40]. The transduction and cultivation of presorted populations does not take into account cellular interactions and may introduce artifacts by variations in culture conditions. In contrast, the transduction of bulk populations considers cellular interaction but does not enable the manipulation of the target cell population when non-selective LVs are used. The Ad-LV technology allows for the selective manipulation of target cells present in complex, bulk suspensions of different cell types. Thus, the Ad-LV technology offers the advantage that functional differences in CD4+ and CD8+ CAR-T cells can be studied under the same culture conditions. Accordingly, variations in cytokine secretion or marker expression can be attributed to the CD4/CD8 ratio of the CAR-T product. 

As previously published, our analysis confirmed the equal cytolytic activity of CD4+ and CD8+ T cells [33]. In line with a publication by Schmuek-Henneresse et al., a tendency toward the enhanced secretion of IFN-γ was observed with higher frequencies of CD8+ CAR-T cells, while IL-2 secretion dropped in the absence of CD4+ CAR-T cells [32]. This is in contrast to studies by Sommermeyer et al. and Xhangolli et al., who described higher IFN-γ secretion by CD4 + CAR-T cells or no differences in cytokine secretion between CD4+ and CD8+ CAR-T cells, respectively [31,40]. This discrepancy may be a result of differences in the CAR architecture, analysis timepoint, cytokine quantification method, and tumor model. Additional studies will support the dissection of the role of CD4+ and CD8+ CAR T cells in tumor lysis and persistence. Of note, the study of different cellular subsets in mixed populations using Ad-LVs is limited to subtypes that can be identified using surface markers. 

This study presents data on a novel system for selective genetic modification in vitro. However, targeted LVs are also under evaluation in the field for in vivo manipulation but face certain limitations [18,19,26]. In general, MV-LV titers are lower as compared to VSV-G pseudotyped lentiviral vectors, and for in vivo transductions, high vector doses would be required. The need to develop high-productivity systems for use in vivo is further underlined as transduction enhancers such as Vectofusin-1 cannot be applied. In addition, due to vaccination or previous infections, population immunity against measles is high. Consequently, if applied directly to the patient, neutralizing antibodies will reduce the transduction efficiency of Ad-LVs. Alternative pseudotypes that are not neutralized in human serum, such as LVs pseudotyped with Nipah envelope proteins, could be evaluated for this type of technology. However, it has already been shown that directly targeted Nipah-based vector systems require a lower distance between cellular antigen and the LV particle membrane [14]. This indicates that additional components, such as adapters, might affect the functionality and flexibility of Ad-LVs when placed in the context of the Nipah pseudotype. In addition, the in vivo generation of CAR T cells requires the transduction of quiescent T cells. Although this has been shown to be feasible for targeted MV-LVs, the transduction of resting T cells with Ad-LVs still remains to be investigated.

Due to its ease of application and broad flexibility, the Ad-LV technology may support the development of optimized cellular therapies. In addition, the technology can be adapted for the clinical production of CAR-T cells ex vivo. Transduction efficiency in both cell subsets could be precisely controlled and may thereby be adjusted to a patient’s situation. In addition to CAR-T cells, Ad-LVs will be useful tools for gene function studies and the characterization of other clinically relevant cell types. 

## 5. Conclusions

In summary, we generated a highly flexible and selective targeting system for LVs, which allows the efficient transduction of cell lines and primary cells, demonstrating the potential of this technology. Compared to other versatile targeting systems, we provide the most detailed analysis of selectivity using different transduction protocols and target cells. Due to their flexibility, Ad-LVs may not only be interesting for CAR-T cell therapy but may be adapted for various clinical and experimental settings.

## 6. Patents

N.C., J.M., T.S., and A.K. have relevant IP to the findings disclosed.

## Data Availability

The datasets presented in this study are available from the corresponding author on reasonable request.

## Supplementary Materials

The following supporting information can be downloaded at: https://www.mdpi.com/article/10.3390/v14102157/s1, Figure S1: Titration and productivity of Ad-LVs; Figure S2: Evaluation of transduction enhancers; Figure S3: Stability of gene transfer; Figure S4: Production of CAR-T cells using the CD20-CAR-encoding Ad-LV.
