# Supplementary material for: Adapter-Mediated Transduction with Lentiviral Vectors: A Novel Tool for Cell-Type-Specific Gene Transfer
Source: Viruses. 2022 Sep 30;14(10):2157. doi: 10.3390/v14102157 (PMC9607492; doi:10.3390/v14102157)
Supplement: Supplementary file 1 [file viruses-14-02157-s001.zip › viruses-1918527-supplementary.pdf]

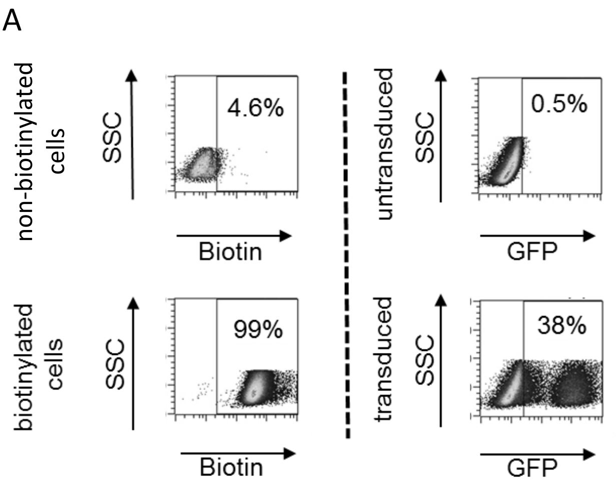

B

LV Batch	Titer (TU/ml)	
	Ad-LV	Ad-LV (opt)
1	5.85 x 10^5	1.69 x 10^6
2	6.99 x 10^5	2.28 x 10^6
3	9.09 x 10^5	3.41 x 10^6

Figure S1. Titration and productivity of Ad-LV.
To determine LV titer and thereby productivity Ad-LV particles were titrated on biotinylated cells. (**A**) Representative data of randomly biotinylated HT1018 cells confirming successful biotinylation by staining with fluorescently labeled α-biotin antibody. Subsequently, Ad-LV was titrated on biotinylated cells. (**B**) Titers determined from three independent LV productions of Ad-LV and Ad-LV(opt).

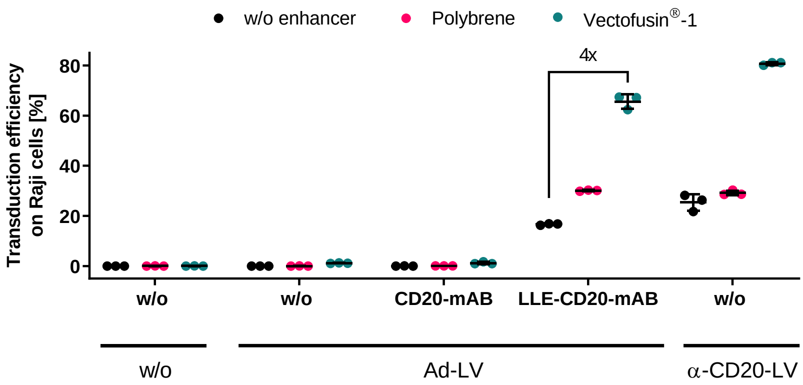

Figure S2. Evaluation of transduction enhancers.
Evaluation of the transduction enhancer polybrene and Vectofusin®-1 to increase efficiency. Transduction efficiency of Raji cells was investigated in absence (w/o) or presence of an α-CD20 antibody (1000 ng/ml) that was non-biotinylated or biotinylated using the Ad-LV (0.05 TU/cell). A direct targeted α-CD20 of Ad-LV was used as positive control.

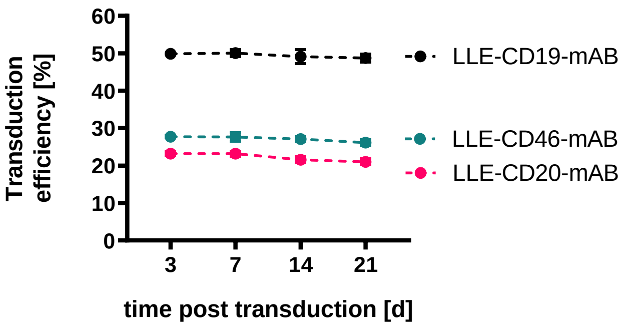

Figure S3: Stability of gene transfer.
Raji cells were transduced in presence of either an LLE-CD19-mAB, LLE-CD20-mAB or an LLE-CD46-mAB (Figure 3B) and the ratio of GFP-expressing cells was determined over a period of 21 days. The ratio of GFP expressing cells was stable over time confirming lentiviral mediated transgene expression**.**
Data are represented as mean ± SD of 3 technical replicates.

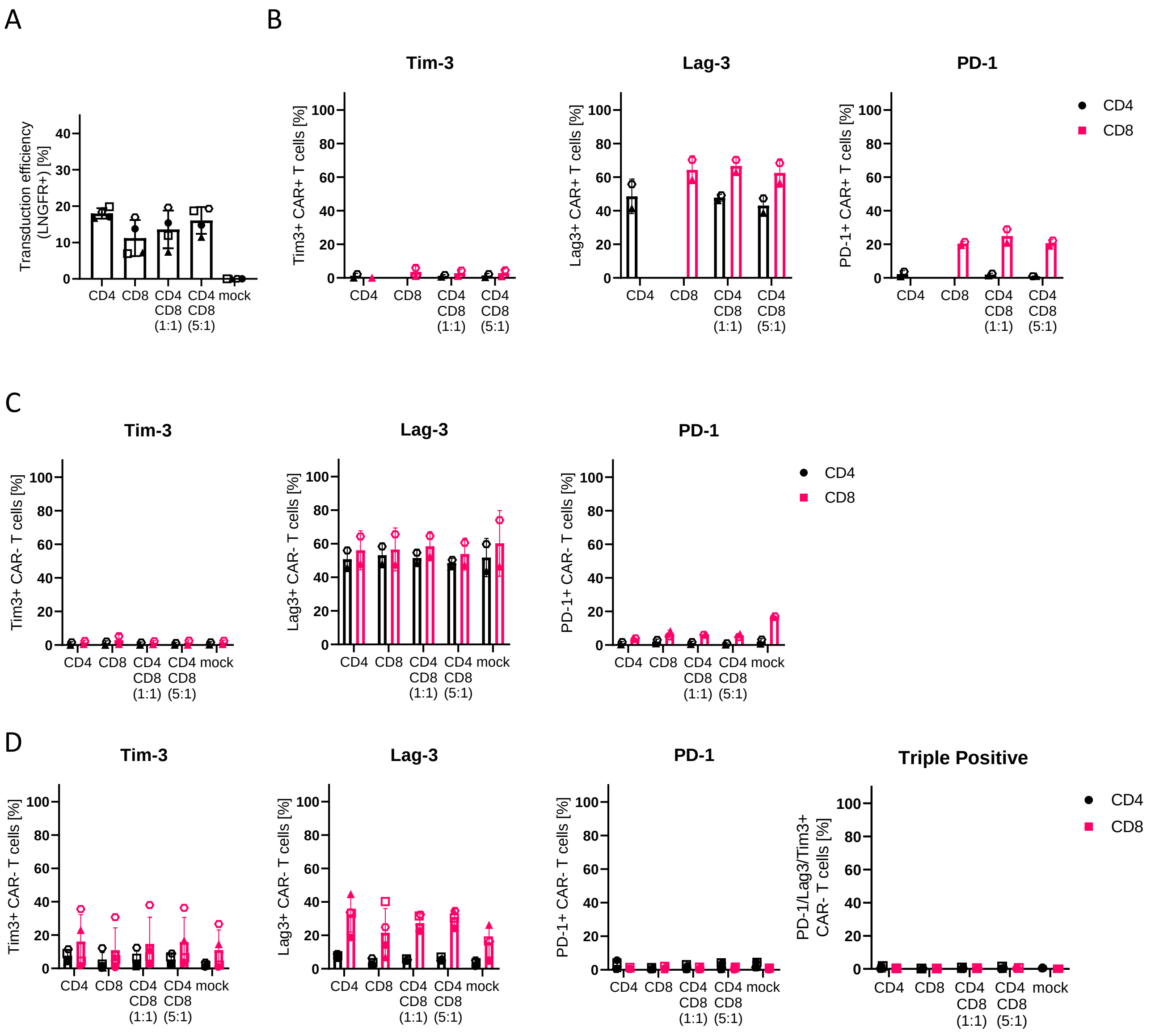

Figure S4. Production of CAR-T cells using the CD20-CAR encoding Ad-LV.
CAR-T cells with varying CD4/CD8 ratio were generated from activated Pan T cells using Ad-LV. (**A**) Transduction efficiency was determined by quantification of LNGFR marker expressing cells on CD3+ T cells one day before co-culture. Activation/Exhaustion marker expression was analyzed 6 days post setup of the co-culture assay. (**B**) Exhaustion marker expression on CAR T cells before co-culture. (**C**) Exhaustion marker expression on non-transduced T cells before co-culture. (**D**) Exhaustion marker expression on CAR-negative T cells after 6 days of co-culture. Data are represented as mean ± SD of 2-4 different donors.
